# Access to health services by lesbian, gay, bisexual, and transgender persons: systematic literature review

**DOI:** 10.1186/s12914-015-0072-9

**Published:** 2016-01-14

**Authors:** Grayce Alencar Albuquerque, Cintia de Lima Garcia, Glauberto da Silva Quirino, Maria Juscinaide Henrique Alves, Jameson Moreira Belém, Francisco Winter dos Santos Figueiredo, Laércio da Silva Paiva, Vânia Barbosa do Nascimento, Érika da Silva Maciel, Vitor Engrácia Valenti, Luiz Carlos de Abreu, Fernando Adami

**Affiliations:** Faculty of Medicine of ABC, Príncipe de Gales Avenue, 821, ZIP Code: 09060-650 Santo André, SP Brazil; Regional University of Cariri, Luiz Antônio Street, 1.161, ZIP Code: 63100-00 Crato, CE Brazil; Faculty of Juazeiro, São Miguel Street, 1.224, ZIP Code: 63010-210 Juazeiro do Norte, CE Brazil; Lutheran University Center of Palmas, Teotônio Segurado Avenue, 1501, ZIP Code: 77019-900 Palmas, TO Brazil

**Keywords:** Homosexuality, Comprehensive health care, Health services, Health services accessibility

## Abstract

**Background:**

The relationship between users and health services is considered essential to strengthen the quality of care. However, the Lesbian, Gay, Bisexual, and Transgender population suffer from prejudice and discrimination in access and use of these services. This study aimed to identify the difficulties associated with homosexuality in access and utilization of health services.

**Method:**

A systematic review conducted using PubMed, Cochrane, SciELO, and LILACS, considering the period from 2004 to 2014. The studies were evaluated according to predefined inclusion and exclusion criterias. Were included manuscripts written in English or Portuguese, articles examining the Lesbian, Gay, Bisexual, and Transgender population’s access to health services and original articles with full text available online.

**Results:**

The electronic databases search resulted in 667 studies, of which 14 met all inclusion criteria. Quantitative articles were predominant, showing the country of United States of America to be the largest producer of research on the topic. The studies reveal that the homosexual population have difficulties of access to health services as a result of heteronormative attitudes imposed by health professionals. The discriminatory attendance implies in human rights violations in access to health services.

**Conclusions:**

The non-heterosexual orientation was a determinant factor in the difficulties of accessing health care. A lot must still be achieved to ensure access to health services for sexual minorities, through the adoption of holistic and welcoming attitudes. The results of this study highlight the need for larger discussions about the theme, through new research and debates, with the aim of enhancing professionals and services for the health care of Lesbian, Gay, Bisexual, and Transgender Persons.

## Background

The right to health is considered universal, resulting from a big political mobilization of society. Health care, as a right and duty of the State is, however, an ideal. The reality is that many countries are crossed by frames of exclusion and violation of fundamental human rights, especially for minority social groups such as Lesbians, Gays, Bisexuals, and Transgenders (LGBT).

Reflecting a new form of expression of sexuality, homosexuality is a condition in which a individual seeks sexual, erotic, and emotional satisfaction with someone of the same sex [1]. The word ‘homosexual’ was created in 1868 and although the word ‘heterosexual’ was created and used only in 1930, heteronormative behaviors overlap the forms of homosexual expression [1]. In response to this situation, the LGBT movement has been consolidating worldwide to denounce violations of human and social rights related to the homosexual population, and claim equal rights, especially for access to health services, free of prejudice and discrimination [2].

Studies reveal that members of the LGBT group are more susceptible to health problems, such as abuse of alcohol, tobacco and illicit drugs, obesity, unprotected sex, mental disorders, Sexually Transmitted Diseases (STDs) as HIV/AIDS, bullying, and cervical and breast cancer, as well as violent behavior [1–3]. Although this is a multifactorial scenario, it may be further complicated because of the poor access to health care and the discriminatory practices of involved professionals stemming from homophobia [3–5]. The fact is that experiences of discrimination and prejudice against sexual minorities can directly contribute to a poorer health status [1].

Social stigmatization imposed on sexual minorities have encouraged the recent increase in scientific studies around homosexuality and its relationship with the process of health and disease [6]. Issues such as vulnerability to STD as HIV/AIDS, health conditions, preventive practices, and access to health care strategies have been included in the long list of subjects and objects of research in the field of gender, health, and sexuality studies [3].

In response to the LGBT social movement, some countries, Brazil among them, began to consider the specific needs of this group. To this end, the formulation of public health policies began, such as the Brazil without Homophobia Program, in 2004, and the preliminary version of the National Comprehensive LGBT Health Plan, in 2010 [2]. Despite the fact that advances were identified, one still observe the difficulties faced by LGBT people in accessing the health system as a result of prejudicial and discriminatory behavior, often adopted by health professionals.

It is important to know what touches, what ails, and what affects the homosexual population’s health. Besides biomedical and epidemiological information on disease prevalence, risk, and vulnerability, it becomes important to know the formulation of public health policies directed to the group, implications of gender issues, the structuring of health services, and performance of professionals, since these make up the factors that directly interfere with access and that guarantee the right to health of the homosexual population [6, 7].

Therefore, this study aimed to identify the difficulties associated with homosexuality in access and utilization of health services through the bibliographic survey of scientific literature on the matter.

## Methods

### Stages of review

Systematic review, as PRISMA recommendations, that ran through the steps: (1) defining the research question; (2) establishment of goals; (3) demarcation of inclusion and exclusion criteria; (4) defining the information to be extracted from selected articles; (5) analysis of the results; and (6) data discussion and presentation.

### Research question and objective of the review

The following question informed the review guideline: ‘What are the implications of homosexual orientation for access to health services?’ In line with this question, articles that correlated homosexuality and access to health services were identified.

The following databases were consulted: Scientific Electronic Library Online (SciELO), Literature Latin American and Caribbean Health Sciences (LILACS), The Cochrane Library (Cochrane), and US National Library of Medicine National Institutes of Health (PubMed - NCBI). The following Medical Subject Headings (MeSH) terms were used in the search: ‘homosexuality’, ‘comprehensive health care’, ‘health care’, and ‘health services accessibility’. To increase the scope of the search, Boolean operators AND and OR were used in the following order: MeSH 1 AND MeSH 2 AND (MeSH 3 OR MeSH 4). The option to choose MeSH 3 or MeSH 4 was due to the similarity of their meanings, thereby enabling the identification of a bigger amount of articles on the theme. For all the databases, the same search strategy was adopted.

### Collection period, inclusion and exclusion criteria for sample selection

The collection period occurred from July 2013 to May 2014. Articles published between 2004 and 2014 were surveyed. The analysis followed the predetermined eligibility criteria. The inclusion criteria were: articles published in the selected databases; manuscripts written in English or Portuguese; articles on the LGBT population’s access to health services; and original articles with full text available online. The exclusion criteria were: research reports published on non-scientific websites; studies in the modality of literature review and comments; not original articles, such as editorials, opinions, preface and brief communication; and essays on access to LGBT health services as a result of HIV/AIDS, the latter criterion justified by the fact that the discrimination directed to patients with the disease is a still present phenomenon in society, regardless of sexual orientation.

### Definition, analysis and discussion of extracted information

Two reviewers extracted information from the included studies using a standardized form. The matrix includes information on authors, year of publication, journal database, description of the study sample, the adopted method and the conclusions obtained. The results of the studies were compared, allowing a comprehensive discussion of this topic.

## Results

The initial electronic search in the databases resulted in a total of 667 references. The articles were evaluated according to their year of publication and relation to the theme, resulting in an initial exclusion of 574 articles.

**Fig. 1:**
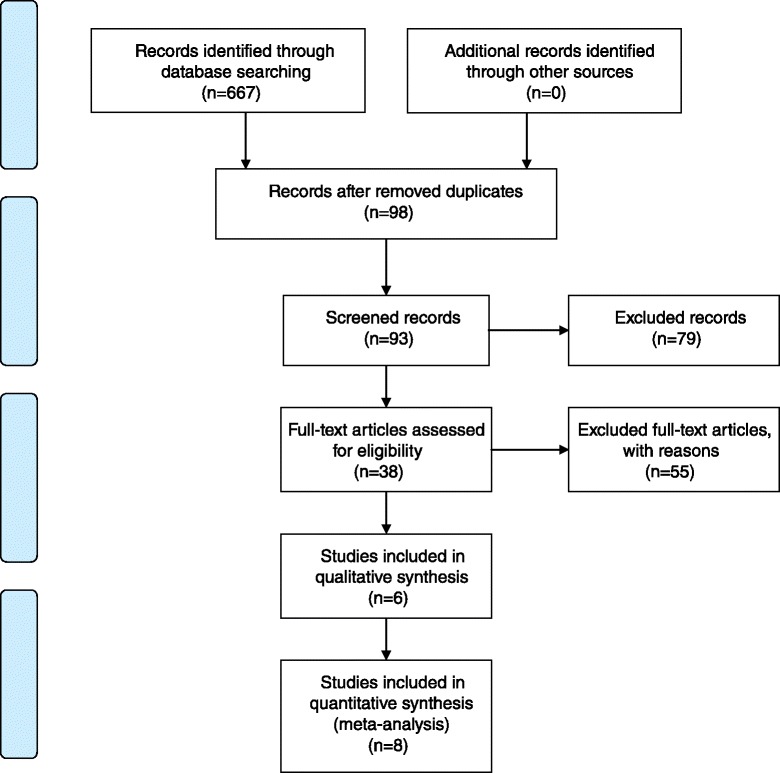
Preferred Reporting Items for Systematic Reviews and Meta-Analyses (PRISMA)

The remaining 93 articles were compared against the other inclusion and exclusion criteria. A total of 14 studies (Fig. 1) met these criteria and formed the final sample.

Data were extracted from each article to obtain structural information and content, identify year and place of publication, research methodology, number of study participants, and main results (Table 1).

**Table 1:** Summary of publications that addressed accessibility to health and health care services

Publication/country	Database	Population	Obtaining results	Key findings
Qualitative Study				
Melo, Perilo, Braz and Pedrosa. [1] Brazil	SciELO	52 managers in Health and 43 LGBT activists	Analysis of government documentary sources and application of semi-structured interviews.	There are difficulties in implementation of health actions with respect to homosexuals, which implies the search for services only in situations of emergency care, considered the gateway system by LGBT group
Barbosa and Facchini [2] Brazil	SciELO	30 homosexual women	Ethnographic observation and application of semi-structured interviews.	Due to be instances of discrimination against homosexuals in health services, LGBT members seek health care generally in situations of greater illness.
Araújo, Saraiva, Galvão and Albuquerque. [3] Brazil	SciELO	One homosexual woman	Experience report through a case study with application of semi-structured interview.	There is weakness in interpersonal relationships between women and health professionals, because of homosexual orientation, with communication difficulties and disattention to issues linked to the experience of sexuality.
Gutiérrez [4] Mexico	LILACS	92 homosexuals (49 men and 43 women)	Application of semi-structured interviews.	Homosexual women have needs for sexual and reproductive health that cannot be contemplated because of discriminatory and prejudicial barriers related to sexual orientation on the part of health services.
Seaver, Freund, Wright, Tija and Frayne. [8] USA	PubMed	22 homosexual women	Accomplishment of focus groups with Application of semi-structured interviews.	Healthcare professionals should adopt behaviors inviting the LGBT population, assuring them the information that should take into account all aspects of health and not only aspects related to sexuality.
Boyce, Barrington, Bolanõs, Arandi and Paz-Bailey [9] Guatemala	Cochrane	8 transsexuals, 16 homo/ bisexual men and 5 heterosexual men	Application of semi-structured interviews.	Gay and bisexual men suffer from difficulties of access to health services due to homosexual orientation as well as experience breach of confidentiality and discrimination in services by professionals.
Quantitative Study				
Buchmueller and Carpenter [5] United States	PubMed	5,265 homosexuals and 802,659 heterosexuals	Application of structured interviews and accomplishment of logistic regression.	There are differences in attendance between homosexual and heterosexual women with regard to access to insurance and health services; the first present more difficulties, because of prejudice and discrimination based on sexual orientation.
Heck, Randall and Gorin. [6] United States	PubMed	614 homosexuals and 93,418 heterosexuals	Application of structured interviews and accomplishment of logistic regression.	Important differences were found in access to health care, especially for gay women, because of heteronormative attitudes of the services.
Kerker, Mostashari and Thorpe. [10] USA	PubMed	19,349 between homosexuals and heterosexuals	Population-based cross-sectional surveys and application of structured interviews.	Homosexual women have more difficulty accessing health services due to discriminatory attitudes in services, which implies difficulty of being up-to-date with their routine exams, such as the Pap smear and mammogram.
Hiestand, Horne and Levit. [11] USA	PubMed	516 women among lesbians and bisexuals	Snowball sampling technique and application of online structured questionnaire.	Lesbians and bisexual women have more health risks than heterosexual, particularly diseases affecting the genitals, because they have limited access to services as a result of prejudice and discrimination on grounds of sexual orientation present in the attitudes of the area professionals.
Steele, Tinmouth and Lu [12] England	PubMed	489 homosexual women	Self-administered survey with application of structured questionnaires.	Disclosure of sexual orientation in health care reflects the difficulty of access and use of health services, which implies not seeking care early and increased susceptibility to health disorders.
Hoffman, Freeman and Swann. [15] USA	PubMed	733 young homosexuals	Application of online structured questionnaire and accomplishment of focus groups.	The young LGBT population reveals the need for health professionals to provide health care not only in reducing sexual risk (by associating, culturally, sexual diseases to the homosexual population), but also in promoting health in family disputes.
Goldsen, Kim, Barkan, Muraco and Ellis. [14] USA	PubMed	96,992 participants (homosexual, bisexual and heterosexual) over 50 years	Application of online structured questionnaire.	The LGBT population over 50 years experiences major challenges to reveal their sexual orientation in health services. This, coupled with the stigma of aging, it raises the barriers to care services, resulting in the onset of diseases on a larger scale.
Herrick, Stall, Chmiel, Guadamuz, Penniman, Shoptwan, Ostrow and Plankey. [13] USA	PubMed	6,972 gay men	Prospective study with recruitment and application of structured interviews.	The internalized homophobia by the homosexual hinders the search for health services, bringing him a set of health problems, among them those of mental order.

Source: SciELO, Lilacs, PubMed, and Cochrane

Legend: LGBT - Lesbians, Gays, Bisexuals and Transgenders

**Fig. 2:**
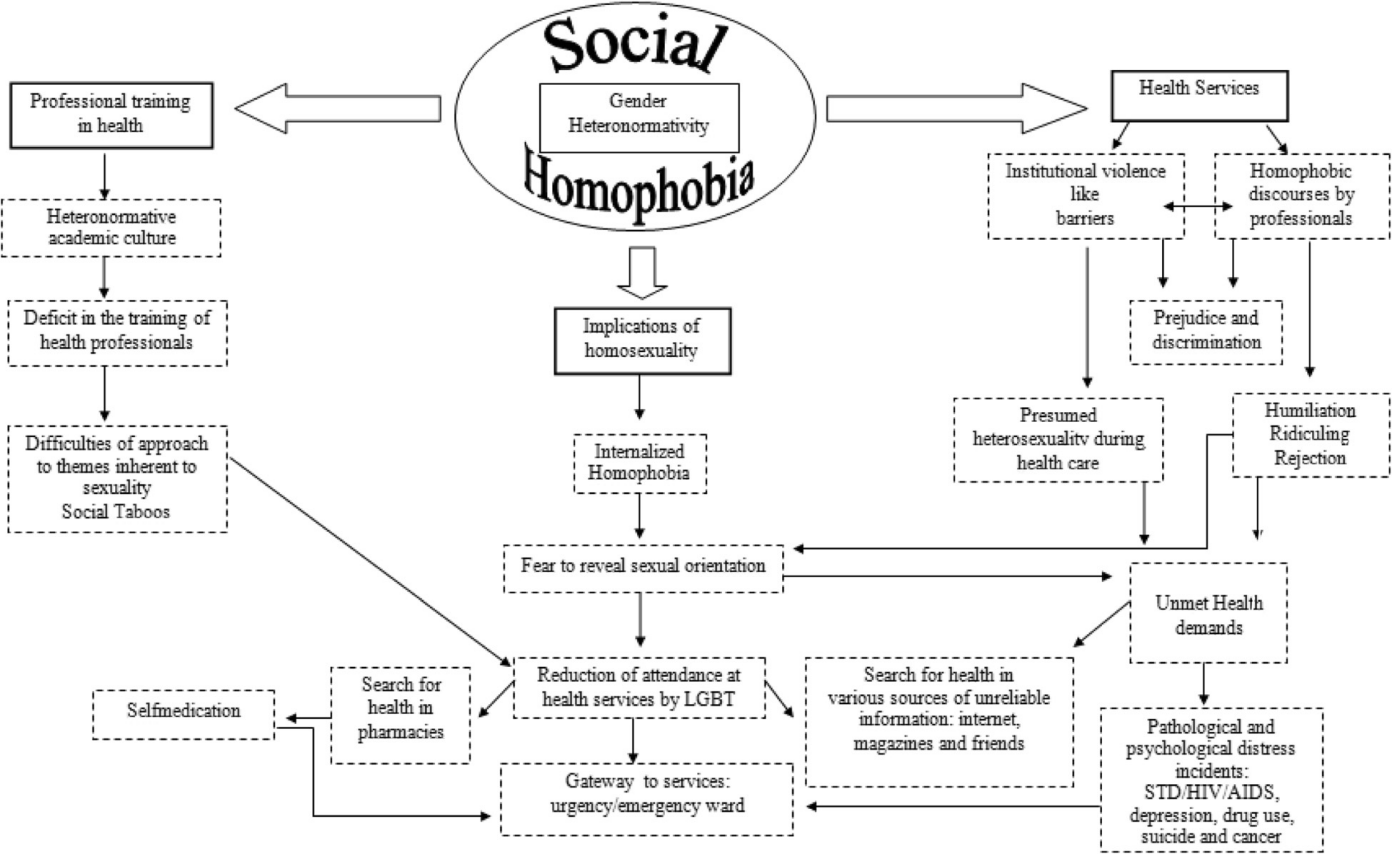
Axles considered obstacles for access to health services and health care. LGBT: Lesbians, Gays, Bisexuals, and Transgenders.  Themes.  Developments of Themes.  Bidirectional flow implications

Figure 2 presents a flowchart that summarizes the main conclusions about the studies found, analyzing the interface between them.

The identified studies revealed the main implications of homosexuality towards access to health services: differences in health care between heterosexual and homosexual individuals, particularly for the female homosexual population [5–10]; communication difficulties as a accessibility barrier to the gay population to health services [3]; prejudicial conduct adopted by health professionals [4, 11]; breach of confidentiality during consultations [9]; disclosure of sexual orientation in health services [12]; persuit of health services in major conditions situations, because of institutional homophobia [1, 2]; internalized homophobia [13]; aging and homosexual orientation as access barriers [14]; need for holistic care beyond the sexual issues of the homosexual population [8]; and higher performance of professional services towards the care of LGBT youth [15]. These findings are discussed in categories that gather the main conclusions of research.

In order to understand the LGBT population’s health, several models may be adopted as parameters to examine how identities and cultural and social arrangements influence the access to health care, status and results of sexual minorities. Accordingly, the main conclusions of the studies were grouped into three axes based on two models usually adopted in research involving sexual minorities regarding health disparities imposed on the group.

The axis called “implications of homosexuality” is based on the “minority stress model”. This model starts from the premise that sexual minorities experience the chronic stress resulting from the suffered social stigmatization, with negative impacts on health [16]. According to this model, proximal stress processes include internalized homophobia (self-directed aversion), and stigma (expectation or fear of being rejected in society, with concealment of sexual orientation) [16]. These conditions, found in the conclusions of the studies, may justify the absence of demand for health services.

The axis called “health services” and “vocational training in health” worked on the premise of the model of “social ecology”. This model recognizes that environmental factors act upon the health determinants of a population [17]. With regard to homosexual health, the model is useful to conceptualize that the social behavior of the group (non-heterosexual orientation) affects the environment and, in turn, is affected by it. Accordingly, social structures such as family, teaching, religion and society are influenced by the social behavior of sexual minorities, and also influence the group’s life, for example, the health care. This relationship may justify the findings of the studies, where the vocational training in health is grounded on a heteronormative and prejudiced culture, which implies an institutional violence in health services, which can consequently justify the reduction of health care by the LGBT population.

In order to do the contextualization of the selected studies, the axes “health services”, “implications of homosexuality” and “vocational training in health” were renamed in the form of categories and discussed with the aim of explaining the results of research and discussing their findings, respectively in: (i) homophobia in health care: LGBT access barriers; (ii) implications of homosexuality in self-care and health services access; and (iii) vocational training in health: deficits to attendance for the LGBT population.

In the first category, the challenges for health care to the LGBT population are discussed, which, in part, are associated with sexual practices and lifestyles of the group perceived as deviant from a supposed normal range defined by gender relations, which indicate heterosexuality as dominant pattern of sexual orientation. The second category explores the discriminatory attitudes on the part of health professionals, when referring to health care for LGBT clients. The last category addresses the behavior of health professionals when caring for sexual minorities, with such behavior being partly influenced by stereotypes, social taboos and myths about non-heterosexual sexual orientation.

## Discussion

### Homophobia in health care: LGBT barriers to access health services

In the item on the sexuality and sexual/erotic practices, the gender relations impose the role of subject/active on the man, and the role of the object/passive on the woman, who should be attracted, owned and dominated by the first. This condition reinforces social patterns of sexual experience, thereby defining guidelines to sexual/erotic practices and hailing heterosexuality, or in other words, the emotional/sexual attraction to the opposite sex, as dominant pattern of sexual orientation [1].

When these papers are rejected, in the example of homosexuality, rejection behaviors are envisioned as a vicious circle, transmitted from generation to generation, and characterized as homophobia [1]. Homophobia can be defined as the rejection, fear or irrational intolerance towards homosexuality [7, 18].

Research conducted in 102 municipalities with a sampling of 2,363 respondents found that 89 % of participants were against male homosexuality, and 88 % against lesbianism and bisexuality in women [4], reinforcing the view that homophobia is largely socially determined [2].

Nevertheless, a study conducted by The Association of Gays, Lesbians, Bisexuals, and Transgenders Parade in Brazil, in 2006, with 846 members of the group, found that 67 % had suffered discrimination because of homosexual orientation and that 59 % had experienced some type of physical violence [2]. Although such studies do not represent the entire population, they are an important indicator of the existence of homophobia, which pervades the daily life of the LGBT population.

Homophobic discourses are present in the conduits and in the minds of health professionals. For some area workers, the LGBT population is a group of sick people, not worthy to formalize marriages and adopt children; by witnessing attitudes of affection between members of the group, the repulsion of these workers was awakened [12].

Misconduct, constraints, prejudiced connotations or even verbal abuse on the part of professionals in health facilities, generate reduction in attendance and in seeking assistance. These attitudes can be experienced as violent situations (sometimes silent and sometimes concrete) that may contribute to the deviation of own body care and the health of the LGBT population [4, 6].

As a result of this reality, the group has fears revealing their sexual orientation in health services, anticipating the negative impact that such an attitude can generate in the quality of care [3]. As a result of the non-disclosure, the LGBT population is treated as straight and proves to be dissatisfied with the care received, since, in part, this does not address their real needs or even desires [10].

The presence of internalized homophobia within the LGBT population also appears to be another aggravation for them not to search for services [13]. Shame and fear of reprisals after disclosure of sexual orientation have shown association with a set of problems among gay and bisexual men, including depression and anxiety, relationship problems, sexual compulsion, and the use of psychoactive substances [19].

In general, the existence of internal and external homophobia implies the displacement of the population, in cases of illness, to pharmacies first. The LGBT population turn to health units only when the resolution becomes unsuccessful [4]. Self-medication allows the appearance of diseases, with consequent search for units and emergency wards, often considered the gateway to the system [1].

### Implications of homosexuality in self-care and in access to health services

Although most scientific studies have female participants, because historically women seek attendance for health care, knowledge about access to services by the general homosexual population is a key dimension to the formulation of appropriate public policies.

The search for health services for homosexual women compared with heterosexual women reveals a lower frequency in conducting preventive and routine tests, such as preventive examination against cervical and breast cancer [2, 6, 10, 11]. Homosexual women are ten times more likely not to have and/or receive the results of Pap tests and are four times more likely do not to undergo mammography [10].

The reduction in the frequency of performing the Pap smear is justified by homosexual women in the way the examination is conducted, since it can reveal the presence of self-reported physical attributes as masculinized and make it possible to identify a sexuality that may be seen as deviant [2]. In a study of 19,349 participants, between hetero- and homosexuals, lesbians had negative experiences in gynecological clinics, encountering inappropriate reactions and rejections from professionals [10].

Another point that implies the non-procurement of sexual and reproductive health services for gay women is the fact that they do not believe they are at risk of acquiring or being capable of transmitting sexual diseases, since they consider this is only possible in heterosexual relationships and by promiscuity [2]. Still, gay women have reduced protective factors for breast and ovarian cancer, especially those who do not want or intend to become pregnant [10].

Comparatively, gay men also have difficulty accessing health services. A study of 29 participants in Guatemala revealed that gay men have low demand for services and when they seek them their medical needs are unmet as a result of discriminatory attitudes of professionals [9]. It is noteworthy that the AIDS epidemic has raised the pursuit of this population to these services, making gays more likely to seek preventive care for situations that put them at risk of HIV infection [3, 5], as there is a historical and cultural association between male homosexuality and HIV.

With regard to the search for access to health information, a study conducted in Mexico and Brazil, with a sample of 122 homosexual participants, revealed that this group’s sources of information are gay friends, magazines/books, websites, and civil society organizations [2, 4]. Among older gay women, the main source of information, in most cases, is other gay women, preferably the sexual partner, causing the prevention of certain diseases to be seen as ‘the couples business’ and not as something that should be shared with a health care professional [2], which increases susceptibility to aggravations.

Information obtained improperly and not making a precocious quest for health care favor the appearance of other problems. Connection with consumption of alcohol, tobacco and drugs, suicide attempts and depression tendency, arise with high frequency in the LGBT [3–5] population, as well as problems related to sexual and reproductive health. Many homosexuals, by not revealing their sexual orientation and playing a typical role within the genre to which they belong, are more likely to develop psychological disorders [20], especially young homosexuals, due to the difficulty experienced with social and family acceptance [15, 21, 22].

A survey of 733 LGBT youth in the United States identified the need for greater sensitivity on the part of health professionals, in an attempt to solve the problems of this population [15]. Young LGBT affirmed the importance of a more comprehensive look at the area of workers on health promotion, brokering conflicts, especially in the family, and the reduction of social homophobia [8].

Similar data are found in the elderly homosexual population, who experience major challenges and barriers in access to health services, to reveal their sexual orientation. The stigma is associated with the believing that aging and homosexuality raises the risk of social isolation, poor physical and mental health, cognitive impairment, and mortality in the general elderly population [14].

### Vocational training in health: deficits in attendance by the LGBT population

The perception of homosexuality as a universal moral and the consequent rejection directed at homosexuals, constitute some of the greatest difficulties for the health professional in LGBT customers’ approach [1, 3]. Criminalization and stigmatization of homosexuality are important barriers to providing access and utilization of services by health professionals [23].

Research conducted in the United States with 116 students in health area, 75 % female, found that 8-12 % believed that homosexuality should be punished; 5–12 % of students disliked sexual minorities; and 51–53 % noted that homosexuality was against their religious beliefs [24]. In England, of a group of 137 students, in 83 % heterosexual women, 16 % felt uncomfortable if they were responsible for the care of a homosexual person [24].

In the Middle East, a survey of 126 students, 87 % heterosexual women quantified the degree of homophobia with the adoption of an index (the Gay Index). This index has a scale of 25 items with a total score of zero to 100, where values above 50 indicate strong inclination to homophobia. Although the results reveal indices below 50, there were still indications that homophobia was present at the students’ academy, with negative implications for the provision of health care to the LGBT population in short, medium, and long term [22].

Inexperience and/or deficit in education, capacity, and professional conception during academic training may also be considered an obstacle to the service group. Still, the thematic approach inherent in sexuality that often goes against professional modesty prevents satisfactory health care being directed to the group [9].

Intervention strategies, such as continuing education, can be adopted to prepare health professionals for non-discriminatory service directed at the LGBT group, granting the right to comprehensive care, as provided in the legislation. Continuing education experiences reveal satisfactory results. In Kenya, a 2-day training of health professionals provided information on men who have sex with men, their sexual risk behaviors, and their health needs. The post-training evaluation, 3 months after this intervention, found a reduction in prejudice attitudes and increased knowledge of these health professionals regarding the particular health of this population [25].

For both, there is the need to provide, in the training of health professionals, evidence-based clinical information relating to the health care process facing the LGBT population. These requirements are: communication patterns; understanding the relationship between health, illness, and gender issues; sensitive approach to the homosexual patient; and addressing the most common health problems [24]. It is pertinent to reduce the difficulties of accessibility to health services, as well as violation of confidentiality and discrimination on the part of professionals, by the adoption of these attitudes [9]. Even health professionals criminalize homosexuality. Instead, they can be encouraged to provide a supportive and safe environment in which sexual minorities can discuss their risk behaviors, sexuality, and health problems [23].

The universality of the right to health requires the proposal of strategies and specific attention, according to the singularities of subjects seeking services. This implies that social determinants, such as sexual orientation and gender identity, should be known and cultivated by health professionals. It is hoped that the problematization of homosexuality can advance, so that the LGBT population has their rights of citizenship respected, especially with regard to the field of integrated health.

## Conclusions

From the data obtained, it was possible to initiate a reflection on the weakness that still exists between homosexuality and the attention given by health services. Despite the presence of qualitative studies, considered a limitation of this review, before the subjective nature of its methodological approach, the review highlights the prejudice and discrimination suffered by the LGBT population in accessing good quality health services.

The LGBT population experience difficulties communicating with health professionals, apart from the fear of assumptions about their sexual orientation, and of embarrassing situations when expressing their homosexuality/bisexuality, due to the homophobia present in professionals’ conduct.

The exclusion and marginalization in health services implies a reduction in attendance and the subsequent search for assistance, contributing to the deviation of this clientele, in view of their own body care and reducing the chance of developing educational and preventive health work.

It becomes necessary therefore, to ensure that, apart from the provision of qualified and equipped health services, there are trained professionals stripped of discriminatory attitudes in that area. These should be able to analyze the health status of their clients, taking into consideration the health, social, and cultural context in which they are placed. To that, the accomplishment of new research on the theme may provide a broader discussion and generate favorable changes in the health care of the LGBT public.

For this, important measures are: the introduction of this topic into undergraduate curricula of health professionals; conducting training with already working professionals; monitoring the implementation of laws addressing social homophobia; and the development of empowerment strategies across the LGBT population, so that it is possible to act in the relentless pursuit of their rights, becoming visible as subjects of their own history.

## Abbreviations

Cochrane: The Cochrane library
HIV: Human immunodeficiency virus
STD: Sexually transmitted diseases
LGBT: Lesbian, gay, bisexual and transgender
LILACS: Latin American and Caribbean Literature on Health Sciences
MeSH: Medical Subject Headings
PubMed: US National Library of Medicine, National Institutes of Health
SciELO: Scientific Electronic Library Online
